# Clinical Outcomes and Prevalence of Sarcopenia in Patients with Moderate to Severe COVID-19

**DOI:** 10.3390/jcm11216578

**Published:** 2022-11-06

**Authors:** Shuhei Yamamoto, Yasunari Sakai, Keiji Matsumori, Ryuji Osawa, Shun Ito, Daichi Tsukakoshi, Tomoki Ohno, Hiroaki Ohta, Takashi Ichiyama, Masamichi Komatsu, Yosuke Wada, Masayuki Hanaoka, Shota Ikegami, Hiroshi Horiuchi

**Affiliations:** 1 Department of Rehabilitation, Shinshu University Hospital, Nagano 390-8621, Japan; 2 Department of Intensive Care Unit, Shinshu University Hospital, Nagano 390-8621, Japan; 3 First Department of Internal Medicine, Shinshu University School of Medicine, Nagano 390-8621, Japan

**Keywords:** COVID-19, rehabilitation, sarcopenia, activities of daily living

## Abstract

Background: The purpose of this study was to investigate the effectiveness and clinical outcomes of inpatient rehabilitation for patients with severe COVID-19 in Japan. Methods: Patients with severe COVID-19 who underwent rehabilitation during hospitalization were included. The Medical Research Council (MRC) score and short physical performance battery (SPPB), such as physical function assessment and the intensive care unit (ICU) mobility scale, the functional status score for the ICU, and Barthel index as activities of daily living (ADLs) were evaluated at admission and discharge or transfer from the hospital. The correlation between SPPB at discharge and each factor at admission were also analyzed. Furthermore, the prevalence of sarcopenia was evaluated by defining SPPB of <9 points at discharge as sarcopenia. Results: The median age of the total of 23 patients was 59 years (interquartile range (IQR): 47–67), 73.9% were male, and the median PaO_2_/FiO_2_ at admission was 172.0 (IQR: 123.0–209.0). All physical function and ADL parameters were significantly improved from the time of admission to discharge (*p* = 0.014 for the MRC score and *p* < 0.001 for all others). Moreover, SPPB at discharge significantly correlated with WBC (Spearman’s rho = −0.473, *p* = 0.041), C-reactive protein (Spearman’s rho = −0.468, *p* = 0.044), and exhibited a significant trend with PaO_2_/FiO_2_ (Spearman’s rho = 0.429, *p* = 0.067) and age (Spearman’s rho = 0.409, *p* = 0.083). Although the median Barthel index at discharge was 90 points, 47% of patients had sarcopenia as defined by an SPPB of <9 points. Conclusions: Early rehabilitation for patients with severe COVID-19 improved physical function and ADLs during hospitalization. However, 47% of patients had the same level of sarcopenia at discharge.

## 1. Introduction

During the COVID-19 pandemic, cases of various complications such as mental and cognitive dysfunction have been reported in patients with severe SARS-CoV-2 infection due to prolonged stay in the intensive care unit (ICU) [1,2,3]. Patients with COVID-19 infection can present with severe symptoms such as acute respiratory distress syndrome, septic shock, acute renal failure, and thromboembolism [1,4]. These patients are admitted to the ICU and require mechanical ventilation, extracorporeal membrane oxygenation, and renal replacement therapy in addition to the usual treatment. During pandemics, neurological complications due to prolonged ICU stays are well known, with ICU-acquired weakness (ICU-AW) being the most frequent and 38% of patients having physical weakness after 1 year [2]. Patients hospitalized with COVID-19, especially those who are critically ill, almost always present with respiratory symptoms, including ARDS [4]. In patients with respiratory disease who do not have COVID-19, the greater the severity of their hospitalization, the greater the loss of mobility. Previous studies have demonstrated that this loss of mobility can be improved with rehabilitation interventions [5,6].

However, there are limited studies examining the effects of inpatient rehabilitation for patients with COVID-19; only a few therapists have experience in this type of rehabilitation because rehabilitation is performed with strict infection control measures during the COVID-19 pandemic. Although acute rehabilitation for patients with COVID-19 is occasionally recommended, the appropriate subjects and intervention methods are a subject of discussion [7,8]. Depending on the policy of the hospital, some hospitals do not provide rehabilitation interventions for patients with COVID-19. Reports from the United States indicate that for patients with COVID-19, inpatient physical therapy with increased frequency and duration is directly associated with better mobility at discharge and a higher probability of discharge to home [9]. Nonetheless, only very few reports have investigated the clinical outcomes of COVID-19, particularly in more severe patients who underwent rehabilitation interventions in Japan, especially from a single institution. As our hospital received the first COVID-19 patient in February 2020, we have treated primarily severe cases requiring respiratory management and intensive care [10]. Although multicenter studies allow the analysis of several cases, they also have drawbacks in examining the effects of rehabilitation because of the lack of uniformity in rehabilitation protocols. However, validation at a single institution makes it easier to evaluate the effects of rehabilitation even with a relatively small number of cases.

The purpose of this study was to investigate the effectiveness and clinical outcomes of inpatient rehabilitation for patients with moderate to severe COVID-19 in Japan.

## 2. Materials and Methods

### 2.1. Study Population

This was a retrospective study of patients with moderate to severe COVID-19 who were admitted to Shinshu University Hospital. In our practice area, patients with mild to moderate COVID-19 were admitted to a local secondary emergency hospital, and moderate to severe COVID-19 patients, who had PaO_2_ below 60 mmHg without oxygen and required ICU management, were admitted to Shinshu University Hospital. As our institution is a tertiary care hospital, all patients are those who are determined to be difficult to manage intensively in secondary care. All patients with COVID-19 had a positive PCR test result for SARS-CoV-2. After admission, the patients were first examined by a rehabilitation physician, followed by respiratory rehabilitation intervention by rehabilitation staff, specifically physical and occupational therapists with expertise in the respiratory system. Patients who died during hospitalization and patients outside the country were excluded from this study. All patients were able to live independently before the onset of COVID-19. The research protocol was approved by the ethics committee of the hospital (approval number 5499). Study information, including the objectives, inclusion and exclusion criteria, and primary outcome, was published in the publicly available University Hospital Information Network (UMIN-CTR, unique identifier: UMIN000047293).

### 2.2. Characteristics of Patients

We collected the following data from medical records: age; gender; body mass index; comorbidities; mechanical ventilation; PaO_2_/FiO_2_, PaO_2_, PaCO_2_, and pH at hospital admission; duration from COVID-19 onset or hospital admission to start of rehabilitation; hospital stay; discharge to home or transfer to another hospital; and laboratory data.

### 2.3. Rehabilitation Program

Rehabilitation staff with experience in pulmonary rehabilitation were trained to manage patients with COVID-19 by wearing appropriate personal protective equipment. The pulmonary rehabilitation program was conducted according to a multidisciplinary program described in an Italian position paper [7]. The type, intensity, timing, and methods of intervention were tailored to individual patients according to age, clinical severity, mechanical ventilation status, and comorbidities, beginning with a progressive exercise program for 20 min daily for 5 days per week. For patients on mechanical ventilation, chest physiotherapy, lung expansion procedures, and mobilization in bed, including limb resistance training, sitting, and foot-stomping were performed. Patients without mechanical ventilation received rehabilitation such as gait, balance training, standing, and sitting exercises, and resistance training for all four limbs. Furthermore, patients with a high level of physical autonomy were trained on a cycle ergometer with low-intensity exercise, with or without mechanical ventilation. The initial training workload was chosen to start at 0 and gradually increase until the patient-rated dyspnea and/or leg fatigue as 4 or 5 on a modified 10-point Borg scale. To avoid the risk of environmental contamination, chest physiotherapy, including bronchial hygiene using self-administered disposable instruments, and lung expansion procedures were performed as necessary.

### 2.4. Assessment of Physical Functions and Activities of Daily Living (ADLs)

Rehabilitation staff evaluated the following physical functions and ADLs at hospital admission and discharge:

#### 2.4.1. Physical Functions

We evaluated the Medical Research Council (MRC) score and short physical performance battery (SPPB) as markers of physical function. The MRC score is a muscle strength assessment with a score of 0–5 points for shoulder abduction, elbow flexion, wrist dorsiflexion, hip flexion, knee extension, and ankle dorsiflexion, with a total score of 60 points [11]. The SPPB is a multidomain performance assessment that consists of three domains, viz., balance test, standing test, and 4-m walk test. Each domain is evaluated on a scale of 0–4 points, with a total of 12 points [12].

#### 2.4.2. ADLs

The intensive care unit mobility scale (IMS), functional status score for the ICU (FSS-ICU), and Barthel index were evaluated as indicators of ADLs. The IMS is a simple scale that evaluates ADLs from lying in bed to walking in 10 categories [13], based on whether the patient is able to do so or not. The FSS-ICU is a scale that evaluates five domains from turning over to walking on a scale of 0–7 points depending on the amount of assistance, with a total of 35 points [14]. The Barthel index is a scale that evaluates basic ADLs such as eating, dressing, toileting activities, and bathing according to the amount of assistance, with a total score of 100 points [15].

### 2.5. Statistical Analysis

Data are expressed as mean and standard deviation for normal distribution and as median and interquartile range for nonnormal distribution. Categorical data were expressed as numbers and percentages. Differences from admission to discharge were compared using the Wilcoxon rank-sum test and chi-squared or Fisher’s exact test for continuous and categorical variables, respectively. A two-tailed *p*-value of <0.05 was considered to be statistically significant. For all outcome changes from admission to discharge, effect size estimates and 95% confidence intervals were reported. Moreover, correlations between SPPB at discharge, background factors, and laboratory data at admission were analyzed using Spearman’s correlation coefficients. Statistical analysis was conducted using STATA version 15.0 (StataCorp, College Station, TX, USA) and R version 4.0.3 (R Foundation for Statistical Computing, Vienna, Austria; ISBN 3-900051-07-0, URL http://www.R-project.org (accessed on 12 August 2022)).

## 3. Results

### 3.1. Characteristics of Patients

**Figure 1:**
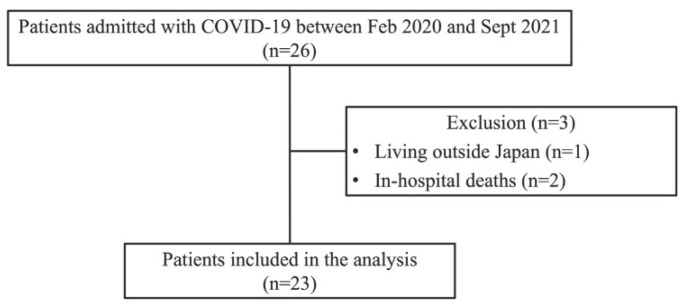
Flow diagram of this study.

A total of 23 patients with COVID-19 were included from February 2020 to September 2021. Among the 26 patients enrolled in this study, 23 (88.5%) fulfilled the inclusion criteria and received intensive treatment in the ICU (Figure 1). Table 1 shows the baseline profiles of the patients. Severe hypoxemia with a median P/F of 172.0 (interquartile range (IQR): 123.0–209.0) was observed in the patients on admission. Moreover, there were 10 patients (43.5%) requiring mechanical ventilation. Among the included patients, the median hospital stay was 13 days (IQR: 10–20 days), and the proportion of discharge to home was 52.2%. The excluded patients (*n* = 2) due to hospital deaths were patients with COVID-19 on extracorporeal membrane oxygenation (ECMO).

### 3.2. Safety of Physiotherapy in Patients with Severe COVID-19: Complications of Physiotherapy in This Series

In this study, no adverse events such as unplanned extubation, accidental arterial catheter or feeding tube removal, uncontrolled arrhythmia, and falls occurred during physiotherapy. Six patients (26.1%) had desaturation of SpO_2_ > 4% at baseline, and one patient (4.5%) had orthostatic hypotension of SBP > 20 mmHg at baseline during rehabilitation. Infection control for COVID-19 among physical therapists is also an important issue, and none of the physical therapists working in the COVID-19 ward were SARS-CoV-2-positive during the study period.

### 3.3. Effects of Rehabilitation on Physical Functions for Hospitalized Patients with COVID-19

**Figure 2:**
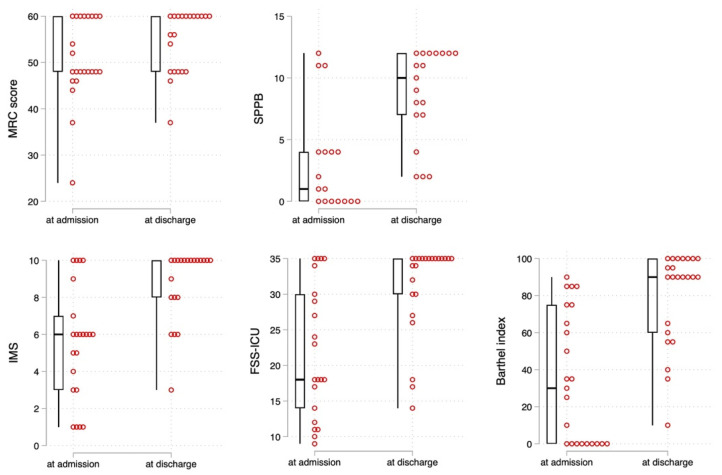
Box and dot plots of changes in physical function and ADL from hospital admission to discharge. MRC = medical research council, SPPB = short physical performance battery, IMS = intensive care unit mobility scale, FSS-ICU = functional status score for the ICU.

**Figure 3:**
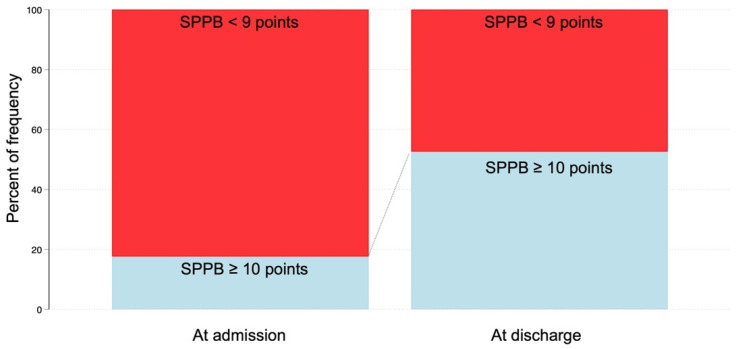
Prevalence of sarcopenia defined as SPPB < 9 points.

Table 2 presents the laboratory data, physical functions, and ADL changes during hospitalization, and Figure 2 depicts the box and dot plots of changes in physical functions and ADLs. The MRC and SPPB scores, which are measures of motor function, improved significantly from admission to discharge (z = −2.44; *p* = 0.014 and z = −3.42; *p* < 0.001, respectively). Similarly, the IMS (z = −3.71; *p* < 0.001), FSS-ICU (z = −3.79; *p* < 0.001), and BI scores (z = −4.19; *p* < 0.001), which are measures of ADLs, improved significantly from admission to discharge based on the Wilcoxon rank-sum test. The median BI at discharge was 90 points. Furthermore, the Asian Working Group for Sarcopenia 2019 (AWGS 2019) criteria [16], which define sarcopenia as an SPPB score of ≤9, were used to investigate the prevalence of sarcopenia at discharge. There were 47% of patients with sarcopenia as defined by the SPPB score who could be evaluated (Figure 3).

**Table 2:** Changes in laboratory data, physical function, and ADLs from admission to discharge.

	Admission (*n* = 23)	Discharge (*n* = 23)	*p* Value	Effect Size (95% CI)
Laboratory data				
White blood cell (×10^3^/μL)	8.21 [4.60, 10.84]	7.94 [4.72, 9.90]	0.345	−0.11 (−0.69 to 0.47)
C-reactive protein (mg/dL)	8.15 [4.04, 10.35]	0.31 [0.01, 0.79]	<0.001	−1.86 (−2.54 to −1.15)
Albumin (g/dL)	3.0 [2.8, 3.3]	3.0 [2.8, 3.2]	0.963	0.01 (−0.57 to 0.59)
AST (U/L)	35.0 [29.0, 54.0]	23.0 [21.0, 31.0]	0.012	−0.56 (−1.14 to 0.03)
ALT (U/L)	39.0 [22.0, 68.0]	52.0 [30.0, 76.0]	0.247	0.31 (−0.27 to 0.89)
D-dimer (μg/mL)	1.4 [1.1, 2.5]	1.3 [0.9, 2.0]	0.169	−0.52 (−1.12 to 0.08)
Creatinine (mg/dL)	0.76 [0.62, 0.98]	0.70 [0.59, 0.98]	0.513	−0.14 (−0.72 to 0.44)
BUN (mg/dL)	19.6 [14.2, 28.7]	21.5 [9.8, 23.4]	0.361	−0.32 (−0.90 to 0.27)
eGFR (mL/min/1.73 m^2^)	78.0 [61.0, 95.0]	80.0 [66.0, 96.0]	0.951	−0.02 (−0.59 to 0.56)
Physical functions				
MRC score (points)	48.0 [48.0, 60.0]	60.0 [48.0, 60.0]	0.014	0.50 (−0.11 to 1.10)
SPPB (points)	1.0 [0.0, 4.0]	10.0 [7.0, 12.0]	<0.001	1.39 (0.65 to 2.11)
ADL				
IMS (points)	6.0 [3.0, 7.0]	10.0 [8.0, 10.0]	<0.001	1.25 (0.59 to 1.90)
FSS-ICU (points)	18.0 [14.0, 30.0]	35.0 [30.0, 35.0]	<0.001	1.14 (0.49 to 1.77)
Barthel index (points)	30.0 [0.0, 75.0]	90.0 [60.0, 100.0]	<0.001	1.46 (0.80 to 2.11)

Values are median (interquartile range). CI, confidence interval. ADL, activities of daily living; ALT, alanine aminotransferase; AST, aspartate aminotransferase; BUN, blood urea nitrogen; eGFR, estimated glomerular filtration rate; MRC, medical research council; SPPB, short physical performance battery; IMS, intensive care unit mobility scale; FSS-ICU, functional status score for the ICU.

### 3.4. Association of SPPB at Discharge with Background Factors and Laboratory Data

**Figure 4:**
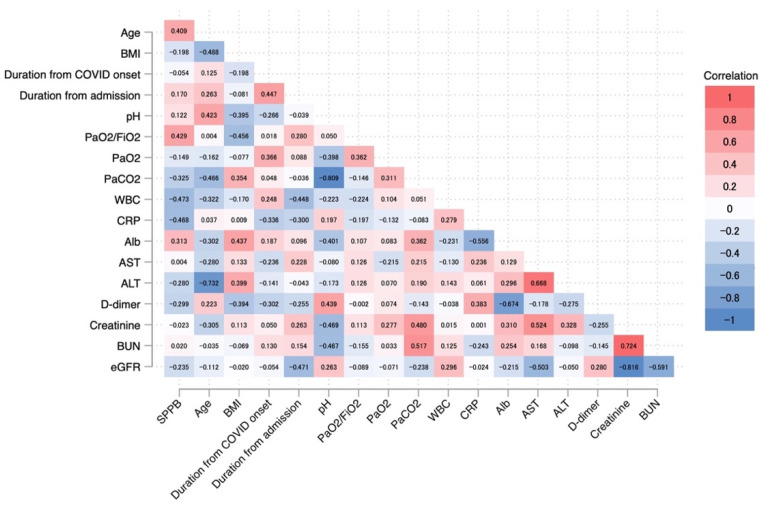
Spearman’s correlations between SPPB and other factors depicted in the heatmap.

The relationship between SPPB scores at discharge and background factors and laboratory data at admission was evaluated using Spearman’s correlation coefficient. The SPPB scores demonstrated a significant correlation with WBC (Spearman’s rho = −0.473, *p* = 0.041) and C-reactive protein (CRP; Spearman’s rho = −0.468, *p* = 0.044), and a significant trend with PaO_2_/FiO_2_ (Spearman’s rho = 0.429, *p* = 0.067) and age (Spearman’s rho = 0.409, *p* = 0.083) (Figure 4).

## 4. Discussion

In this study, we investigated the effect of rehabilitation on hospitalized patients with severe COVID-19 requiring intensive care at a single institution in Japan. Our results demonstrated that patients with severe COVID-19 could improve their physical functions and ADLs after receiving intensive care phase rehabilitation. However, at the time of discharge or transfer from the hospital, 47% of patients were found to have a physical function of SPPB <9 points, i.e., the AWGS 2019 sarcopenia criteria [16]. A previous study from Italy also reported an ICU mortality rate of 44% for patients with COVID-19 admitted to the ICU [17]; however, an extremely important finding of our study was that the overall ICU mortality rate for patients admitted to our ICU was 7% in our hospital. Although patients with COVID-19 on ECMO were excluded owing to in-hospital death, it is remarkable that our hospital provided a phased rehabilitation intervention for patients on ECMO as well [10].

Our study demonstrated that the MRC score and SPPB measured as indicators of physical functions and the IMS, FSS-ICU, and BI measured as indicators of ADLs improved significantly from admission to discharge after rehabilitation in the hospitalization phase in patients with severe COVID-19. A previous multicentric study from Italy published in 2020 reported that pulmonary rehabilitation was implemented in patients with COVID-19, which significantly improved the SPPB and BI scores [18]. Compared with our study, the Italian study had more elderly patients, but the PaO_2_/FiO_2_ was lower in our study (median PaO_2_/FiO_2_: 172.0), and 43.5% of the patients received mechanical ventilation; hence, our study had more patients with severe COVID-19 than the Italian study. To our knowledge, this is the first report to show that physical functions and ADLs improved after rehabilitation in patients with severe COVID-19 requiring intensive care. A previous study from the United States also reported that early rehabilitation for patients with COVID-19 was associated with lower in-hospital mortality (odds ratio: 0.11, 95% confidence interval: 0.06–0.19), even after adjustment for multiple confounding factors and COVID-19 markers. In that previous study, the average time from admission to the start of rehabilitation was 5 days [9], whereas the median time in our study was 3 days. It is possible that the earlier rehabilitation was more effective for patients with severe COVID-19 in our study.

In addition, we investigated the prevalence of sarcopenia, defined as an SPPB score of ≤9. We found that 47% of patients had sarcopenia, although their BI scores improved to 90 points at discharge. A previous meta-analysis showed that the prevalence of sarcopenia in COVID-19 patients was 48%, which is similar to the prevalence in our study [19]. A previous study on patients with severe COVID-19 requiring mechanical ventilation reported that 22% of patients were discharged with a cane or rolling walker and 14% were admitted to a rehabilitation facility, although 94% of patients were functionally independent before admission [20]. The previous Italian study reported that the BI score was 95 points and the SPPB score was 7 points at discharge after rehabilitation for patients with COVID-19 [18]. In the present study, the BI score at discharge after rehabilitation was 90 points and the SPPB score was 10 points, which is similar to the previous study. Although our study included patients with more severe COVID-19 than did the previous study, the higher SPPB score at discharge is a remarkable result. Furthermore, CRP and WBC at admission were associated with factors affecting high SPPB recovery at discharge. Several systematic reviews have reported that higher CRP and D-dimer levels were associated with more severe COVID-19 [21,22], muscle weakness, and exercise intolerance [23]. It was recognized that the greater the severity of COVID-19, the higher the levels of inflammatory markers such as WBC, CRP, and severe respiratory failure, resulting in lower PaO_2_/FiO_2_. The severity of COVID-19 at admission may also affect the physical condition of the patient at discharge and thus the effectiveness of rehabilitation. A previous systematic review reported that exercise, early mobilization, and multicomponent programs improved recovery after ICU admission due to severe respiratory illness and may be generalizable to patients with COVID-19 [24]. The present study reported results similar to that review.

This study has several limitations. First, there is no control group in this study because all patients with COVID-19 underwent the intervention. Therefore, a control group would be needed in the future to evaluate the effectiveness of the rehabilitation. Second, it is difficult to generalize the findings to all patients with COVID-19, including mild cases, because our hospital is a tertiary emergency center and accepts only severe cases. Third, owing to reasons of infection control, we could not perform standard respiratory muscle testing and pulmonary function tests, including assessment of diffusion capacity. Therefore, it is not possible to define the extent to which the decline in physical performance observed at admission was attributable to lung or respiratory muscle dysfunction. Nevertheless, we believe that this is an important study in which physical function assessment was performed to the maximum possible extent within the limited medical resources available. Fourth, the number of cases analyzed was smaller than that of other studies because our analysis was performed at a single institution. However, our study has the advantage of performing a detailed physical evaluation.

## 5. Conclusions

Early rehabilitation for patients with severe COVID-19 admitted to the ICU improved physical functions and ADLs during hospitalization. However, 47% of patients had the same level of sarcopenia at discharge in Japan.

## Figures and Tables

**Table 1:** Baseline patient characteristics.

	Overall (*n* = 23)
Age (years)	59 [47, 67]
Male gender (*n*, %)	17 (73.9)
BMI (kg/m^2^)	24.4 [22.4, 28.6]
Comorbidities (*n*, %)	
Hypertension	7 (30.4)
Dyslipidemia	5 (21.7)
Diabetes	8 (34.8)
Atrial fibrillation	1 (4.3)
Coronary artery disease	4 (17.4)
COPD	1 (4.3)
Interstitial pneumonia	1 (6.2)
Stroke	2 (8.7)
Cancer	2 (8.7)
Mechanical ventilation (*n*, %)	10 (43.5)
Duration of mechanical ventilation, days	4 [1, 7]
pH	7.42 [7.36, 7.47]
PaO_2_/FiO_2_	172.0 [123.0, 209.0]
PaO_2_ (mmHg)	90.5 [75.3, 107.0]
PaCO_2_ (mmHg)	35.4 [31.2, 39.2]
Duration from COVID-19 onset to start of rehabilitation (days)	8.0 [4.0, 11.0]
Duration from admission to start of rehabilitation (days)	3.0 [2.0, 4.0]
Duration of rehabilitation (days)	12 [7.0, 20.0]

Values are mean (standard deviation), median (interquartile range), or *n* (%). BMI, body mass index; COPD, chronic obstructive pulmonary disease; COVID-19, SARS-coronavirus-2 disease 19.

## Data Availability

Not applicable.
